# Gemcitabine-Induced Drug Rash With Eosinophilia and Systemic Symptoms (DRESS Syndrome): A Case Report

**DOI:** 10.7759/cureus.98273

**Published:** 2025-12-01

**Authors:** Sara Ait Elfaqir, Othmane Zouiten, Leila Afani, Mohamed El Fadli, Rhizlane Belbaraka

**Affiliations:** 1 Department of Medical Oncology, Mohammed VI University Hospital, Marrakesh, MAR; 2 Department of Medical Oncology, Faculty of Medicine and Pharmacy, Mohammed VI University Hospital, Cadi Ayyad University, Marrakesh, MAR; 3 Department of Oncology, Mohammed VI University Hospital, Marrakesh, MAR

**Keywords:** chemotherapy toxicity, dress syndrome, drug-induced hypersensitivity, gemcitabine, pharmacovigilance

## Abstract

Drug Reaction with Eosinophilia and Systemic Symptoms (DRESS) is a rare, severe, and potentially life-threatening hypersensitivity reaction characterized by fever, diffuse cutaneous eruption, hematologic abnormalities, most notably eosinophilia, and multiorgan involvement, predominantly hepatic. While classically associated with anticonvulsants, allopurinol, or sulfonamides, this syndrome may occasionally be triggered by antineoplastic agents.

Gemcitabine, a nucleoside analogue with a generally favorable safety profile, is widely used in the treatment of various solid tumors. However, exceptional cases of severe immune-mediated cutaneous adverse drug reactions, including DRESS, have been described.

We report the case of a 46-year-old man treated for nasopharyngeal carcinoma, who developed a febrile maculopapular rash with eosinophilia and hepatic cytolysis following gemcitabine-based chemotherapy. The diagnosis of DRESS was established according to RegiSCAR criteria, and clinical improvement was achieved after discontinuation of gemcitabine and corticosteroid therapy.

This observation emphasizes the importance of early recognition of atypical hypersensitivity reactions during chemotherapy and the need for immediate drug withdrawal to prevent potentially severe complications.

## Introduction

Therapeutic advances in oncology have led to the increasing use of chemotherapeutic agents with more manageable toxicity profiles, among which gemcitabine holds a prominent place. This nucleoside analogue is widely incorporated into treatment protocols for solid tumors such as pancreatic and lung adenocarcinomas, as well as certain sarcomas. Its adverse effects are generally well characterized, predominantly hematologic or infectious, while cutaneous reactions remain rare and usually mild.

However, severe immune-mediated toxicities may occasionally occur. Drug Rash with Eosinophilia and Systemic Symptoms (DRESS) is one of the most serious delayed hypersensitivity reactions, defined by fever, widespread rash, hematologic abnormalities, particularly eosinophilia, and multiorgan involvement, most frequently hepatic. According to a large literature review including 172 cases, DRESS carries a mortality rate of up to 10%, and its diagnosis is now standardized through the RegiSCAR scoring system, which stratifies cases as possible, probable, or definite [1].

While DRESS is classically associated with anticonvulsants, allopurinol, and sulfonamides, it may also be triggered by agents not traditionally considered high-risk. In this context, we report a rare case of gemcitabine-induced DRESS syndrome, highlighting the importance of early recognition of atypical febrile rashes during chemotherapy and the need for prompt management to prevent severe complications.

## Case presentation

A 46-year-old male patient with a history of well-controlled asthma was being followed in our institution for the management of a non-keratinizing undifferentiated nasopharyngeal carcinoma (UCNT), initially revealed by rhinologic symptoms and cervical lymphadenopathy. The disease was staged as T2N2M0, corresponding to stage III according to the AJCC 8th edition classification.

Fifteen days after receiving the first cycle of induction chemotherapy consisting of gemcitabine (1000 mg/m² on days 1 and 8) and cisplatin (80 mg/m² on day 1), with standard premedication including adequate intravenous hydration, 5-HT3 receptor antagonists and dexamethasone for antiemetic prophylaxis, the patient developed an isolated mild cutaneous eruption.

**Figure 1:**
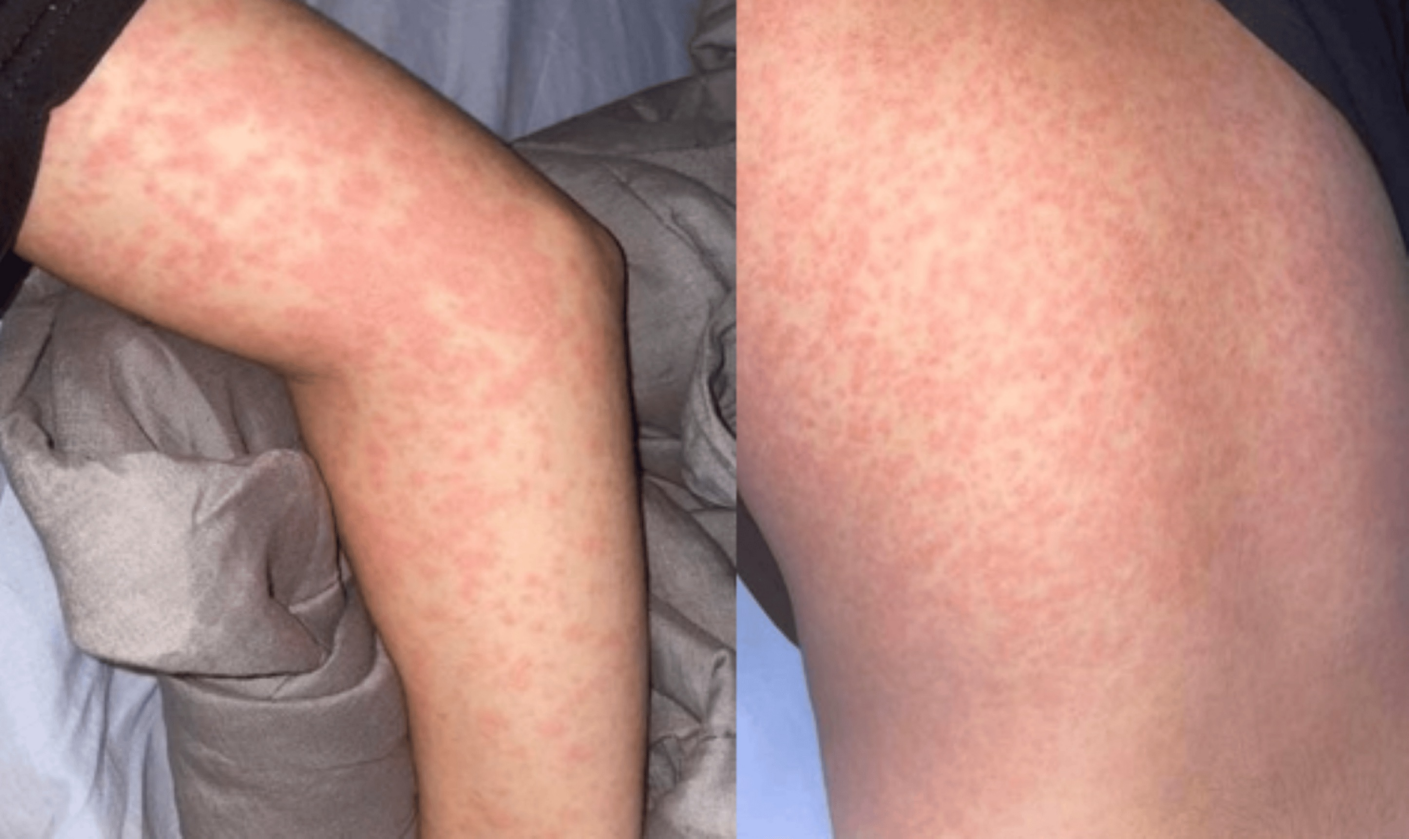
Macular-papular rash

Two days after the administration of the second cycle, he was admitted to the emergency department with a generalized, pruritic maculopapular rash associated with a fever of 38 °C (Figure 1).

In view of the acute febrile exanthema occurring shortly after chemotherapy, an urgent dermatological evaluation was requested. The clinical presentation raised suspicion for a severe drug-induced hypersensitivity reaction, and a targeted laboratory workup was initiated.

The complete blood count revealed eosinophilia at 3500/mm³, associated with lymphocytosis at 5200/mm³. Inflammatory markers were elevated, with a C-reactive protein (CRP) of 78 mg/L. Liver function tests showed acute cytolysis, with ALT at 635 U/L and AST at 480 U/L, while bilirubin and renal parameters remained within normal ranges (Table 1).

**Table 1:** Laboratory test results Hb: Hemoglobin; PLT: Platelets; CRP: C-reactive protein; ESR: Erythrocyte sedimentation rate; AST: Aspartate aminotransferase; ALT: Alanine aminotransferase; GGT: Gamma-glutamyl transferase; ALP: Alkaline phosphatase; TSH: Thyroid-stimulating hormone; ANA: Antinuclear antibodies; Anti-Sm: Anti-Smith antibodies; P24: 24-hour urinary protein excretion.

Biological Parameters	Patient’s Results	Normal values
Lymphocytosis	5200/mm^3^	1500-400mm^3^
Eosinophilia	3500/mm^3^	0-500/mm^3^
Hemoglobin	10.4 g/dL	13-17g/dL
Platelets	476000/mm^3^	150000-400000/mm^3^
CRP	78 mg/L	<6mg/L
ESR	56 mm/h	<20mm/h
AST	480 UI/L	8 – 35 UI/L
ALT	635 UI/L	7 – 45 UI/L
Total Bilirubin	1.2mg/dl	<1.2mg/dL
GGT	48U/L	<55 U/L
ALP	190U/L	40-120 U/L
P24	0.05mg/24h	< 150 mg/24 h
Lipase Level	2U/L	<60 U/L
TSH	0.5 mUI/L	0.4 – 4.0 mUI/L
Urea	0.28 g/L	0.17-0.43 g/L
Creatinine	5.95mg/dL	7-13 mg/dL
ANA	Negative	-
Anti-SM	Negative	-

Serologic tests were negative for HBV, HCV, HIV, and Epstein-Barr virus (EBV) reactivation. Urinalysis and urine culture were also negative.

Given the risk of cardiac involvement described in some cases of DRESS, an urgent transthoracic echocardiography was performed, which subsequently revealed normal findings.

The diagnosis of DRESS syndrome was established based on the presence of multiple major RegiSCAR criteria. The patient presented with a generalized pruritic maculopapular rash associated with fever of 38 °C, marked eosinophilia (3,500/mm³) and lymphocytosis, as well as cervical lymphadenopathy. Liver function tests showed acute cytolysis, with ALT and AST levels about 14 times the upper limit of normal. Viral serologies for HBV, HCV, HIV, and EBV were negative, ruling out infectious causes. No antibiotics such as vancomycin were administered before or during chemotherapy, excluding these as potential causative agents. These findings fulfilled the RegiSCAR criteria for a definite case of gemcitabine-induced DRESS syndrome.

Management was based primarily on the immediate and definitive discontinuation of gemcitabine, which was suspected to be the causative agent. Oral corticosteroid therapy with prednisone was initiated at a dose of 1 mg/kg/day, combined with topical corticosteroids to control the cutaneous lesions. In accordance with best practice, the case was reported to the regional pharmacovigilance center to ensure traceability and contribute to safety data collection.

The clinical course was favorable: the cutaneous eruption progressively improved and completely resolved within five days, with parallel improvement of the general condition and normalization of laboratory parameters. Gemcitabine was not reintroduced. The treatment regimen was modified, and the patient continued chemotherapy with a cisplatin-5-fluorouracil (PF) protocol, in accordance with therapeutic standards for locally advanced nasopharyngeal carcinoma. This regimen was well tolerated, allowing continuation of the oncological treatment without further adverse events.

Overall, the chronology of events, rash onset 15 days after the first cycle, worsening two days after the second administration, and complete recovery within five days of drug withdrawal with normalization of hepatic enzymes after three weeks, was consistent with the delayed immunoallergic pattern typical of DRESS syndrome.

## Discussion

The DRESS syndrome, also referred to as drug-induced hypersensitivity syndrome, is a rare but potentially life-threatening adverse drug reaction. Its estimated incidence ranges from 1 in 1,000 to 1 in 10,000 exposures, with a mortality rate of approximately 10%, most often due to fulminant hepatic failure [1]. Clinically, this syndrome is characterized by a generalized skin eruption, persistent fever, hematologic abnormalities (eosinophilia, atypical lymphocytes), and multiorgan involvement, most frequently hepatic [2,3].

The pathogenesis of DRESS remains incompletely understood. The leading hypothesis involves an idiosyncratic, T-cell-mediated hypersensitivity reaction, resulting in aberrant cytokine secretion. Among these cytokines, interleukin-5 plays a central role by stimulating eosinophil proliferation and activation, accounting for their accumulation in peripheral blood and tissues [4]. Moreover, viral reactivation, particularly of human herpesvirus type 6 (HHV-6), but also EBV and cytomegalovirus, typically occurs two to three weeks after the onset of rash and is considered a distinctive hallmark [5]. Recent studies have also highlighted the role of genetic susceptibility, with specific HLA alleles being associated with an increased risk [6].

Although gemcitabine is generally considered a well-tolerated cytotoxic agent, it may, in rare cases, trigger aberrant T-cell activation or cross-reactivity with endogenous nucleoside structures, leading to immune-mediated hypersensitivity reactions such as DRESS.

The most frequently implicated drugs include aromatic anticonvulsants (phenytoin, carbamazepine, lamotrigine, phenobarbital), allopurinol, sulfonamides (dapsone, sulfamethoxazole, sulfasalazine), minocycline, and certain antiretroviral agents such as abacavir and nevirapine [7,8]. However, the pharmacologic spectrum of DRESS has progressively expanded to include less common agents, notably certain cytotoxic chemotherapies.

Among these, gemcitabine warrants particular attention. This nucleoside analogue, widely used in solid tumors such as non-small cell lung cancer, pancreatic cancer, urothelial carcinoma, breast cancer, and ovarian cancer, is generally well tolerated, with hematologic and hepatic toxicities being the most common and usually reversible [9]. Reported cutaneous side effects are generally mild (transient rash, alopecia), and severe toxidermias are exceedingly rare. Nonetheless, isolated cases of Stevens-Johnson syndrome, toxic epidermal necrolysis, and DRESS have been described [9,10]. In the Gynecologic Oncology Group phase II trial on uterine leiomyosarcoma, a grade-4 rash resolved under prednisone, illustrating the rare but real risk of severe cutaneous adverse reactions to gemcitabine [10].

The diagnosis is based on standardized criteria, particularly those of the RegiSCAR group, which classify cases as “possible,” “probable,” or “definite” according to a scoring system that includes fever, rash, hematologic abnormalities, organ involvement, and prolonged clinical course [1,3,11]. In our patient, the combination of a diffuse maculopapular rash, marked eosinophilia, significant hepatic cytolysis, persistent fever, and exclusion of alternative causes strongly supported the diagnosis of gemcitabine-induced DRESS (Table 2). The latency period between drug initiation and symptom onset in DRESS syndrome typically ranges from two to eight weeks, reflecting its delayed immunoallergic pathogenesis. In our case, the eruption appeared 15 days after the first gemcitabine administration, which remains consistent with this time frame. According to the Naranjo adverse drug reaction probability scale, the causal relationship between gemcitabine and the DRESS syndrome was rated as ‘probable’ (score = 6).

**Table 2:** Major criteria of DRESS syndrome DRESS: Drug Reaction with Eosinophilia and Systemic Symptoms

RegiSCAR criteria	Description
1	Maculopapular rash developing ≥3 weeks after starting with a limited number of drugs
2	Prolonged clinical symptoms ≥2 weeks after discontinuation of the causative drug
3	Fever >38°C
4	Liver abnormalities (alanine aminotransferase >100 U/L)
5	Leukocyte abnormalities (at least one present): a. Leukocytosis (>11 × 10⁹/L) b. Atypical lymphocytosis (>5%) c. Eosinophilia (>1.5 × 10⁹/L)
6	Lymphadenopathy
7	HHV-6 reactivation

Management requires the immediate and permanent discontinuation of the offending drug [1]. In severe cases, systemic corticosteroid therapy is indicated, with a gradual taper to minimize relapse risk [3]. Refractory cases may benefit from intravenous immunoglobulins or cyclosporine, although evidence remains limited to small series. The clinical course is usually prolonged (from several weeks to months), necessitating close multidisciplinary monitoring. Long-term follow-up is also essential, as autoimmune sequelae such as thyroiditis or type 1 diabetes have been reported following DRESS [3].

This case carries two major implications. First, it demonstrates that DRESS syndrome can occur under cytotoxic chemotherapy, thereby expanding the spectrum of causative agents and underscoring that any atypical febrile rash in cancer patients should prompt consideration of this diagnosis. Second, it highlights the importance of active pharmacovigilance in oncology, as each reported case contributes to a better understanding of the syndrome and to optimization of its management.

## Conclusions

Anticancer agents, despite their essential role in oncology, can exceptionally be responsible for severe cutaneous adverse drug reactions, including DRESS. The diagnostic challenge is compounded by the frequent use of multiple concomitant therapies in cancer patients, which complicates the identification of the culprit drug. Given the strong causal relationship with gemcitabine, rechallenge with the same agent should be strictly avoided. Alternative non-gemcitabine-based regimens should be considered in future chemotherapy planning to prevent recurrence of DRESS syndrome. Early recognition of the syndrome and immediate withdrawal of the suspected medication are critical to improving prognosis, as progression can lead to life-threatening visceral involvement. Management requires rapid initiation of supportive and specialized care, often with systemic corticosteroids in severe cases. Finally, re-exposure to the implicated drug is absolutely contraindicated, and systematic reporting to pharmacovigilance systems is essential to enhance collective knowledge of these rare but serious events.
